# Evaluation of the Activation Energy for Pyrolytic Degradation of Poly‐L‐Lactide (PLA) During Artificially Accelerated Aging

**DOI:** 10.1002/bip.23642

**Published:** 2024-12-06

**Authors:** Margarita Reit, Natalie Krug, Jan‐Christoph Zarges, Hans‐Peter Heim

**Affiliations:** ^1^ Institute of Material Engineering, Polymer Engineering University of Kassel Kassel Germany

**Keywords:** activation energy, artificially accelerated aging, biopolymers, kinetic models, poly(lactic‐acid), thermal degradation, thermogravimetric analysis

## Abstract

In the course of this study, the pyrolytic degradation characteristics of three poly(lactic acid) (PLA) types were investigated under inert conditions using dynamic thermogravimetric analysis (TGA) across the temperature range of 23°C–600°C with four heating rates. Specifically, the activation energy and its implications were determined at different stages of degradation. For this purpose, a comparative analysis of various isoconversional methods, including Kissinger, Flynn‐Wall‐Ozawa (FWO), Friedman, and Kissinger‐Akahira‐Sunnose (KAS) was undertaken to evaluate the reliability of each. The results indicate a decrease in thermal stability, indicated by a shift of the derived mass loss curves to lower temperatures, as confirmed by an increased water content and decreased crystallinity of the test specimen during aging. The study also highlights that if crystallinity and moisture content increase moderately, the thermal degradation curves remain unchanged. Additionally, kinetic analyses using mentioned models indicate a multi‐step degradation process of PLA. The activation energy fluctuates with the conversion rate, suggesting complex underlying kinetics. These findings emphasize the need for dynamic adjustment of predictive models for material lifespan. The three PLA types were characterized by Differential Scanning Calorimetry (DSC), moisture absorption measurement and Gel permeation chromatography (GPC).

## Graphical abstract

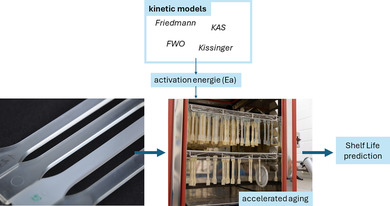

## 1 Introduction

The increasing impacts of climate change have led to a growing interest in sustainable materials across various industrial sectors [1, 2, 3, 4]. Regarding polymers, there's a particular interest in two kinds of polymeric materials; those made from renewable resources (bio‐based) and those that can break down naturally (biodegradable) [1]. Bio‐based polymers are intended to save fossil raw materials, while biodegradable polymers are seen as a potential solution to the gigantic quantities of waste that are already polluting the land and ocean. Although the term “biopolymer” is used for both, it's important to make a difference as not all bio‐based polymers possess inherent biodegradability.

A promising candidate that encapsulates both features is poly(lactic acid) (PLA). This thermoplastic linear polyester can be produced by ring‐opening polymerization from lactic acid out of corn starch or sugar cane, and is completely compostable [1, 2, 3, 4, 5, 6]. The material is non‐toxic, can be biodegraded by the human body and has been approved for food and medical fluid contact [1, 5, 7]. Despite its promising features, PLA is highly sensitive to temperature and humidity [3, 4, 8, 9]. Therefore, the successful integration of PLA into various applications necessitates a comprehensive understanding of its intrinsic properties, especially concerning its degradation processes, to ensure predictability and optimal utilization.

To meet these demands, accelerated aging tests are frequently used [7, 10]. These tests simulate aging by subjecting the material to elevated temperatures over a compressed timeframe. In the field of medical technology, the process of accelerated aging, achieved through temperature elevation, is regulated by the guidelines outlined in ASTM 1980 (Standard Guide for Accelerated Aging of Sterile Barrier Systems for Medical Devices). According to these guidelines the duration of artificially accelerated aging (AAT) is determined by considering factors such as ambient temperature (T_RT_), accelerated aging temperature (T_AA_), real‐time duration (RT) to be simulated, and the Q_10_‐factor. The exact relationship is shown through Equation (1).

(1) $$\begin{array}{c} \text{Accelerated Aging Time}\;\left(\mathrm{AAT}\right)\\ \;=\left(\text{Desired Real Time}\;\left(\mathrm{RT}\right)\right)/\left({Q_{10}}^{\left(T_{\mathrm{AA}}-T_{\mathrm{RT}}/10\right)}\right) \end{array}$$

The Q_10_‐factor, which is derived from Arrhenius' theory, describes that the reaction rate doubles with a 10°C increase in temperature [11, 12]. In standard practice, a Q_10_‐factor of 2 is commonly assumed [12, 13]. However, for a more detailed examination of a specific material, it is advisable to recalculate this factor based on the material's specific activation energy *E*_a_. Equation (2) serves as the mathematical foundation for this recalculation. As shown in this equation, in addition to the activation energy, the universal gas constant R and the temperatures T_1_ and T_2_ are required for the calculation.

(2) $$Q_{10}=e^{\left(E_{a}/R\right)*\left(10/\left(T_{2}*T_{1}\right)\right)}$$

Here, *E*_a_ is the minimal energy required to initiate a chemical reaction, representing the energy barrier that reactants must surpass for the reaction to proceed. In the context of PLA's pyrolytic degradation, *E*_a_ signifies the energy needed to break chemical bonds within the polymer, initiating its decomposition process. Consequently, *E*_a_ provides critical insights into the material's stability and durability, making it a pivotal parameter for evaluating degradation properties.

This study investigates how *E*_a_ varies during aging processes to better understand the influence of different material conditions on storage stability. Since the underlying measurements of the *E*_a_ in this work were carried out using thermogravimetric analysis (TGA) measurements, it was obvious to take a closer look at the aging mechanisms using the TG curves. In polymer science, the analysis of TG or DTG (Derived thermogravimetric) curves is a common means of determining various material properties for example the thermal stability and degradation [14]. Multi‐stage degradation processes can also be identified [14]. This is particularly relevant when additives or compounds consisting of matrix material and filler are involved [15, 16]. Recent approaches, such as that of Dubdub, use the data from TGA measurements together with artificial neural networks to determine the mass fraction from the input data of temperature and heating rate [17].

In concrete terms test specimens of three different PLA types were tested in the freshly molded, pristine and aged state by means of TGA measurements. The DTG curves were compared with each other in order to be able to make statements about the thermal stability. In the following, the correlation between the activation energy and the associated degradation of the three PLA types is discussed. This analytical focus serves as a key component of our exploration, providing insights into the fundamental mechanisms driving the material's transformation over time.

## 2 Theoretical Approach

In model free kinetic methods, which can be isoconversional or isothermal, the activation energy is estimated without assuming a specific reaction model. This approach offers the advantage of determining kinetic parameters without the need to assume fundamental conditions for the specific model [18, 19]. Since a temperature dependency is to be expected from the materials investigated, isoconversional methods are considered in more detail below.

The foundational principle of isoconversional methods bases on the idea that the reaction rate is a temperature‐dependent function at a constant conversion rate α [18, 19]. The *E*_a_ curve provides information about whether the reaction is simple or more complex. For simple processes, *E*_a_ remains practically constant, represented by a horizontal line. However, model‐free kinetics of thermogravimetrically measured reactions rarely yield a constant *E*_a_. If a process involves two (or more) parallel reactions with different *E*_a_, the contribution of the reaction with higher *E*_a_ increases with rising temperature. Therefore, the measured *E*_a_ of such processes increases with temperature and, consequently, with conversion. An increasing activation energy *E*_a_ suggests parallel reactions [19].

### 2.1 Fundamentals of Kissinger Method

The Kissinger method is one of the simplest methods for determining the *E*_a_. The assumption is that the process is a first order reaction [20]. However, it is precisely this assumption that leads to the disadvantages of this method. This prevents the method from recognizing complex reactions and particularities of material degradation. In the procedure for determining the *E*_a_ using this method, the temperature *T*_P_ is determined for each heating rate from the peak α. Accordingly, the *E*_a_ can be derived from the slope of ln(β/T_P_^2^) against 1/*T*_P_.

### 2.2 Fundamentals of Kissinger‐Akahira‐Sunose (KAS) Method

The KAS method is a more advanced version of the Kissinger method. When using the integral KAS method not only one temperature is determined for a maximum value, but a series of experiments conducted at varying heating rates [10, 21]. The peak temperature *T*_p_ is extracted from the TGA measurement curves for each experiment [10, 22]. The *E*_a_ is than determined by plotting ln(${\beta /T}_{p}^{2}$) against $\left(-1/T_{p}\right)$.

### 2.3 Fundamentals of Flynn‐Wall‐Ozawa (FWO) Method

The integral FWO method is an isoconversional technique, which allows to estimate the apparent *E*_a_ according to a conversion rate (α) using thermogravimetric analysis data [19, 21, 22, 23, 24]. In particular, the results of experimentally determined data on the change in sample mass over time at different heating rates are derived [10, 21]. The method involves plotting ln(β) against 1/T, resulting in a linear fit with a slope of −1.052E_a_/R [19, 22, 23, 25].

As described by Pal et al. and Plota et al., the FWO model finds application specifically in polymer systems characterized by the occurrence of multiple reactions simultaneously, leading to changes in *E*_a_ over time. However, this model encounters limitations when significantly different types of reactions occur concurrently [10, 21]. When the *E*_a_ remains independent of α, the FWO method yields values similar to those obtained through the Friedman method [25].

### 2.4 Fundamentals of Friedmann Method

The Friedman method is the most common differential isoconversional approach for determining *E*_a_ without the need to assume a specific kinetic model [22, 24]. This method, similar to the Flynn‐Wall‐Ozawa method, relies on thermogravimetric measurements at various heating rates, making it a versatile tool for studying complex reactions. By plotting ln(dα/dt) against 1/T at different heating rates, the *E*_a_ can be calculated with m = −*E*_a_/R for a given α [19, 22].

## 3 Materials and Methods

### 3.1 Materials

During these investigations, three PLA materials with varying viscosities were analyzed. NP HT 202 and NP HT 203 were acquired from NaturePlast (Mondeville, France), while Luminy L130 was sourced from TotalEnergies Corbion (Gorinchem, Netherlands). All the materials used in this study achieved official certification for food contact applications and are appropriate for processing through the injection molding technique. Table 1 provides detailed information on the relevant characteristics of these materials. It is important to note that no additional additives were compounded into the materials throughout the course of the study.

**TABLE 1:** Bio‐based mass content and melting temperature according to manufacturer's information as well as weight average molecular weight based on own measurements [7].

Material	Bio‐based mass content	Melting temperature *T*_m_	Weight average molecular weight *M*_w_
Luminy L130	100%	175°C	110,783 ± 2191 g/mol
NP HT 202	85%	170°C–180°C	86,617 ± 992 g/mol
NP HT 203	90%	170°C–180°C	113,570 ± 2735 g/mol

### 3.2 Injection Molding Process

To receive a dry and light‐free environment, selected materials were stored in sealed bags. Due to the moisture absorbing nature of PLA, Luminy L130 underwent drying at 100°C for 4 h, while NP HT 202 and NP HT 203 were dried at 60°C for 4 h. This pre‐conditioning process was conducted using a Vacucenter VC 50 vacuum oven (Salvislab, Risch‐Rotkreuz, Switzerland).

Subsequently, the dried materials were employed to produce standardized 1A test specimens (DIN EN ISO 527 [26]) through the injection molding technique. This was carried out using an Arburg Allrounder 270S injection molding machine (Arburg GmbH + Co KG, Loβburg, Germany) with a clamping force of 25 kN and a screw diameter of 22 mm.

For Luminy L130, the injection molding process necessitated a cycle time of 50.8 s and a cooling time of 40 s. NP HT 202 underwent processing with a cycle time of 62.4 s and a cooling time of 50 s. In the case of NP HT 203, the injection molding process demonstrated a notably shorter cycle time of 46.1 s, along with a corresponding cooling time of 2.7 s. The specific temperature profiles utilized during processing are outlined in Table 2. It is noteworthy that, throughout the entire injection molding process, no release agent was utilized, irrespective of the material under consideration.

**TABLE 2:** Temperature profiles used during the injection molding process.

Material	Temperature in°C				
	Zone 1 feeding section	Zone 2	Zone 3	Zone 4 nozzle	mold
Luminy L130	205	205	215	215	40
NP HT 202	175	180	185	185	25
NP HT 203	175	180	185	185	25

### 3.3 Packaging

Fifteen test specimens of each material were collectively packed in a single low‐density polyethylene bag for storage. It's important to note that, in the case of Luminy L130, each test specimen was individually encased to prevent inter‐sample adhesion during the annealing process (Section 3.4). Throughout the aging process, the respective sample bags were stored together in a conventional cardboard box to minimize the impact of light.

### 3.4 Annealing

Due to the intrinsically slow crystallization kinetics of PLA, specimens produced through the conventional injection molding process typically display an amorphous state [27]. In order to increase crystallinity and, consequently, the heat deflection temperature, test specimens of Luminy L130 underwent a 2‐hour thermal treatment at 80°C using an air convection oven manufactured by Memmert (Schwabach, Germany). This thermal pre‐treatment serves not only to enhance crystallinity and stabilize the material [28, 29]. In contrast, NP HT 202 and NP HT 203 are classified as materials with high resistance to heat load, even in their amorphous state. As a result, annealing procedures were deemed unnecessary for these materials.

### 3.5 Increased Temperature Degradation

Accelerated aging was conducted through increased temperature and usual humidity level conditions. For this purpose, the specimens were positioned in a climatic controlled environment set to 50°C and 50% relative humidity.

### 3.6 Thermogravimetric Analysis (TGA)

Kinetic analysis of temperature‐induced material degradation was documented using TGA. For this purpose, TGA‐Modul Q 500 (TA Instruments, New Castle, USA) was employed. In this study, the temperature range of 23°C–600°C was traversed using different heating rates of 5, 10, 15, and 20 K/min, respectively. The measurements were performed with an average sample weight ranging from 9 to 15 mg, in a constant inert atmosphere with a nitrogen flow of 60 mL/min. The software TA Universal Analysis (TA Instruments, USA) was utilized for the analysis of the recorded thermograms. According to the data from this software, Equation (3) was used to calculate the conversion rate where the initial mass is given by ω_o_, ω_T_ denotes the mass at temperature T and ω_f_ represents the residual mass.

(3) $$\alpha =\left(\omega_{o}-\omega_{T}\right)/\left(\omega_{o}-\omega_{f}\right)$$

## 4 Results and Discussion

In this study, three types of PLA (Luminy L130, NP HT 202, and NP HT 203), undergoing various aging periods (no aging, 3 months, 6 months, and 9 months) were examined. The primary focus was on determining the activation energy *E*_a_ for each sample relative to its constitution after aging. To achieve this, the isoconversion methods, outlined in Section 2, were applied. Calculated results are shown in the following Section 4.1, 4.2, with a differentiation between the materials and the distinct aging stages.

As mentioned in Section 3.6, the pyrolytic degradation of PLA in a nitrogen environment was assessed. Supplementary experiments conducted in oxygen exhibited negligible deviations. This observation is consistent with findings reported in the literature. Babanalbandi et al. emphasize that polymer oxidation does not significantly influence its pyrolytic degradation [30].

### 4.1 TGA‐Analysis

**FIGURE 1:**
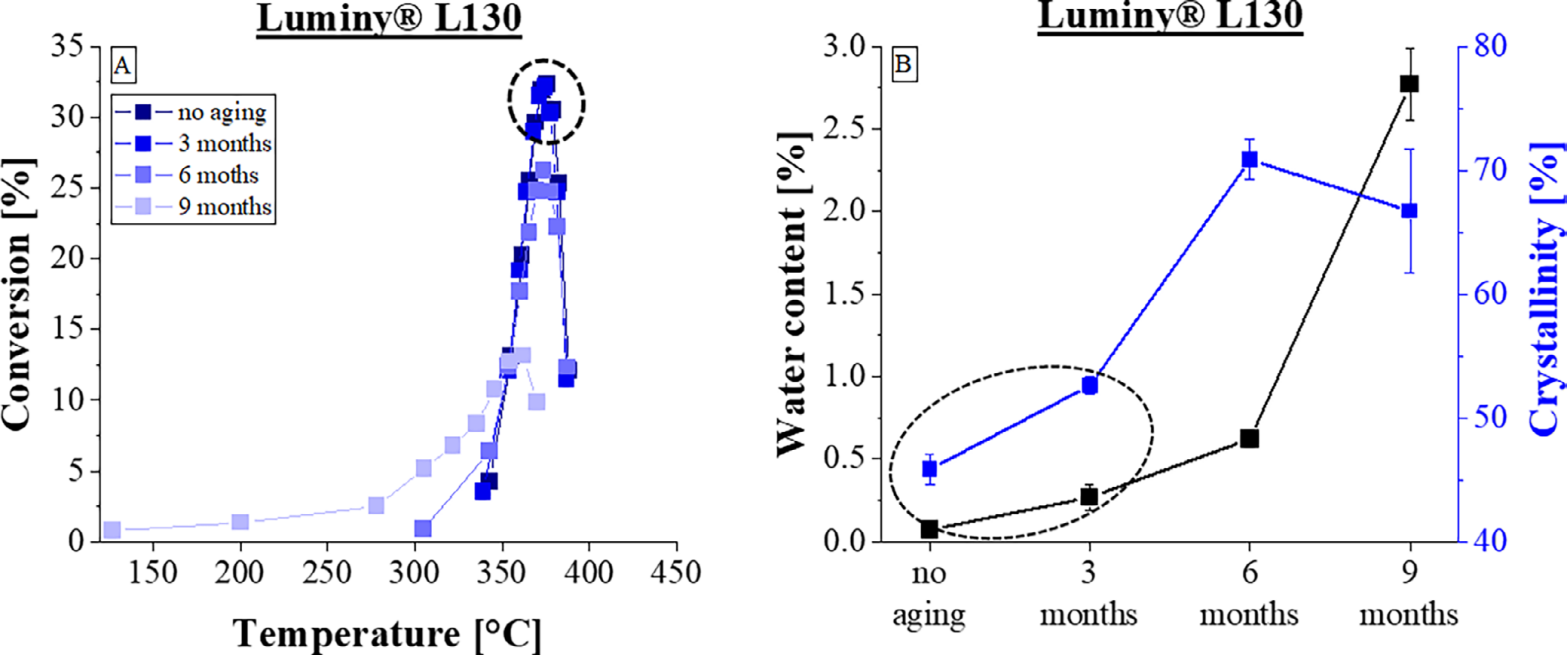
Dependency of conversion rate regarding three aging times of Luminy L130.

Before discussing the results of the calculated *E*_a_, a detailed look at the pyrolysis process of the three PLA types is warranted. Therefore, consideration was given to the derived mass loss observed during TGA measurements. Figure 1A illustrates the correlation between temperature T during measurement and the explicit conversion in % for Luminy L130 regarding the aging times of unaged (0 months), 3, 6 and 9 months. For the representation, the measurement points were recorded starting from an initial mass of 0.95% in 10 steps down to a residual mass of 0.05%. Consequently, the Figure 1A depicts 10 measurement points. For all shown measurements the heating rate was maintained constantly at 10 K/min. An altering of the heating rate β resulted in a displacement of the curve to higher temperatures without a change in the curve profile. This observation suggests that the underlying process is dependent on the temperature rather than being influenced by the selected heating rate [23].

The dehydration process of PLA generally occurs around 100°C. In all three evaluated PLA types this process contributed only a small part to the derived mass loss and is not apparent in all the following figures. The curves of the non‐aged samples and those aged for 3 months exhibit a strikingly similar trajectory starting with a degradation at 340°C. Conversely, the sample aged for 6 months initiates derived mass loss at a substantially lower temperature (305°C). Additionally, the maximum conversion rate value is noticeably lower compared to the before mentioned curves. However, the temperature at which the highest derived mass loss occurs remains consistent across all curves at 375°C. When comparing all previous curves with the most stressed curve, a altered curve progression is observed after 9 months of aging. The degradation process starts considerably below the temperatures of the other test specimen, beginning at 125°C. Also, the maximum conversion rate is lower than at the other aging times.

In summary, the curves demonstrate different starting points of degradation and varying maximum conversion rates, however the basic shape of the curves is very similar, exhibiting a single maximum. To evaluate the effects observed in the derived mass loss diagram, Figure 1B displays the same samples but illustrates the changes in moisture content and crystallinity degree during aging.

It can be observed that during the initial 6 months, the moisture content slightly increases. The crystallinity value experiences a notable increase during this period. However, following these 6 months, there is a decrease in crystallinity, accompanied by a more pronounced increase in moisture content. In Figure 1A,B, areas are marked that are particularly noticeable. These marked circles illustrate the relationship between the crystallinity or moisture content and the conversion. If the crystallinity and the moisture content change to the same extent over the course of aging, as marked round in Figure 1B, this behavior becomes clear in the conversion curves by the fact that the curves behave identically regardless of the storage time. This is also marked round in Figure 1A. This behavior only occurred with the Luminy L130 and in the first 3 months of aging.

**FIGURE 2:**
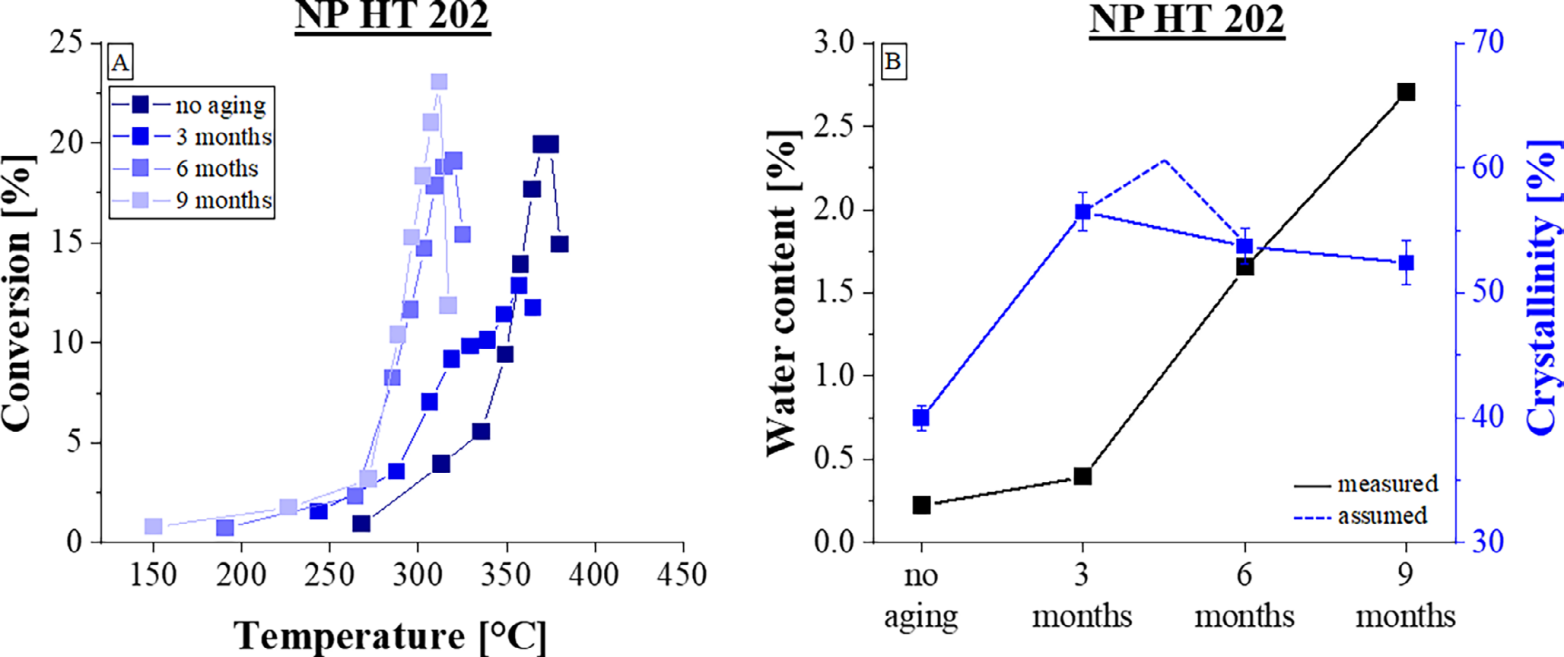
Dependency of conversion rate regarding three aging times of NP HT 202.

The correlation between the pyrolysis process and the structural changes induced by aging for NP HT 202 are discernible in Figure 2. In Figure 2A the derived mass loss curves of all four aging stages lead to different curve trajectories. The unaged sample of the NP HT 202 shows a similar curve to the unaged curve of Luminy L130. It is evident that with increasing thermal stress on the samples, material degradation begins at a steadily lower temperature range while aging. In contrast to Luminy L130, the four curves in Figure 2A do not exhibit the highest mass loss at the same temperature, however, they also exhibit one maximum in the curve progression. The 6 and 9 month aged samples show a similar shift to the left. This indicates that, from this aging time onwards, aged samples begin the highest mass reduction at lower temperatures. The trends in moisture content and crystallinity degree in Figure 2B resemble those of Luminy L130. The moisture content increase over time. It is presumed that the highest crystallinity value occurs between 3 and 6 months of aging. The assumed progression of the crystallinity value is shown schematically in Figure 2B.

**FIGURE 3:**
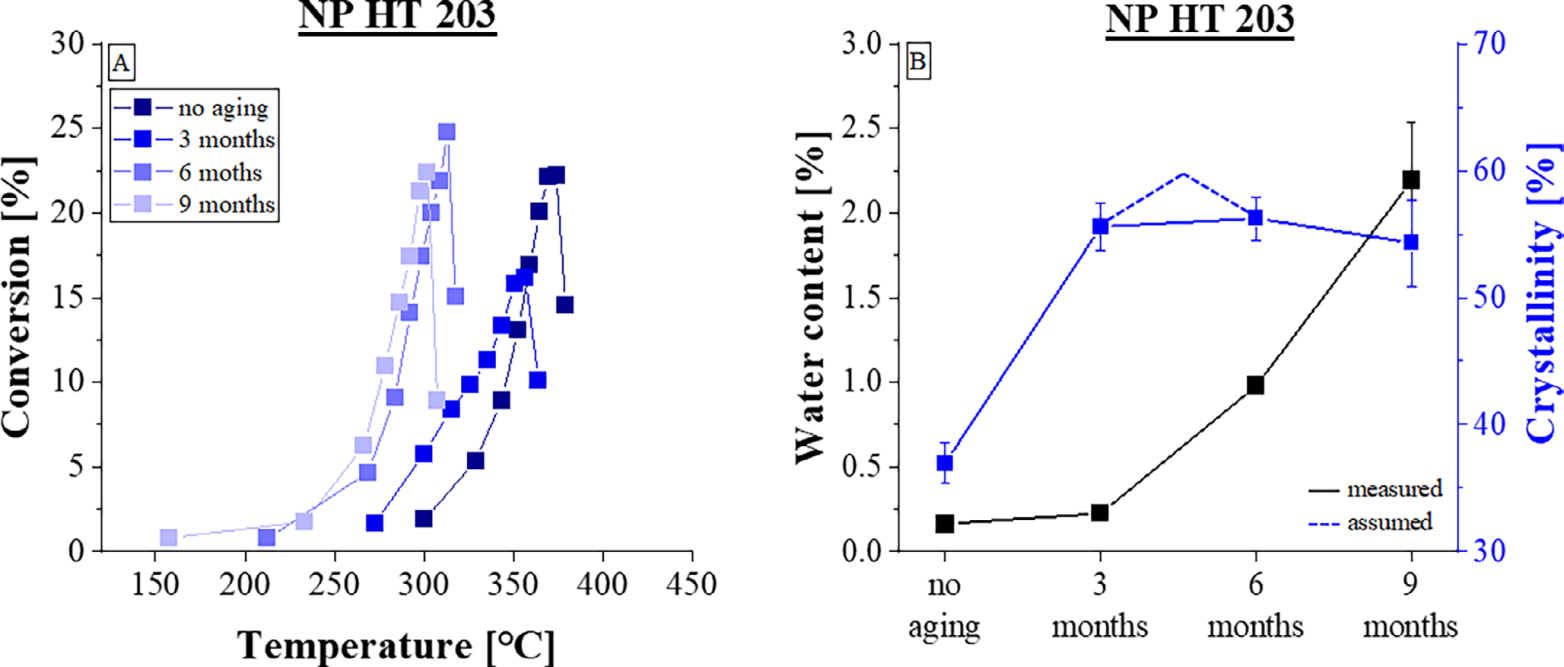
Dependency of conversion rate regarding three aging times of NP HT 203.

The material NP HT 203 exhibits very similar behavior to NP HT 202 as seen in Figure 3A. Here too, the curves show a maximum in the curve progression. The samples aged for 6 and 9 months also show a clear shift to the left towards lower temperatures. The more the test specimens were exposed to the increased temperature, the earlier the mass loss began. Correspondingly, in Figure 3B, showing the moisture content and crystallization degree, similar trends are observed like with the two PLA types evaluated before. As time progresses, the material absorbs moisture, while the crystallization value decreases. Figure 3B also shows the predicted course of crystallinity during aging with a dashed line. It is noticeable that with increasing moisture content, the decomposition process initiates at lower temperatures.

Overall, the investigations of the derived mass loss curves as a function of the aging time showed that the examined thermal stability of all PLA types decreased over the course of the experiments [17]. The shift of the derived mass loss curves to the left towards lower temperatures provides clear evidence of this decrease in thermal stability. On the one hand, this can be explained by the changed degree of crystallization. Crystallinity plays an important role in the degradation behavior of semi‐crystalline PLA [31]. As also shown in Figure 2B, the measurements do not clearly indicate a decrease in crystallinity. However, the documented values were interpreted in light of the literature: Accordingly an increases at the start of aging can be assumed, followed by a rapidly decrease as the aging continues. The crystalline areas break down and the proportion of amorphous areas increases. As a result, the material becomes more susceptible to the effects of aging, as amorphous areas are affected first and predominantly by hydrolysis [18]. This can be explained by the increased water absorption capacity of the amorphous areas in contrast to compact crystalline structures, which make water diffusion more difficult [18]. The increased water absorption was demonstrated in the moisture content measurements. All three PLA types showed the same behavior: As soon as the crystallinity value decreases, the water absorption in the material increases clearly, which indicates that the material is more susceptible to hydrolysis. During the hydrolysis of PLA, the ester bonds are broken down to the components oligomers and monomers [31]. These new degradation products lead to a shift in the curves towards lower decomposition temperatures, triggered by hydrolysis degradation.

At the temperatures applied in this study, the PLA test specimens underwent physical aging. Amorphous PLA for example, only begins to decompose at higher temperatures from 215°C upwards [31]. For all three PLA types, the starting degradation temperature has shifted drastically to lower temperatures after 9 months of aging. It can therefore be stated that the thermal stability has decreased in the meantime.

Another point worth mentioning is that the decomposition of PLA first takes place on the surface when the ambient temperature is below the glass transition temperature *T*_g_ [31]. This means that the degradation processes occur initially on the surface. As described in the results, the surface of the Luminy L130 in particular has changed the most due to the influence of temperature. A slimy layer formed on the surface. It is known that the degradation mechanisms of PLA are both thermal‐oxidative and hydrolytic [16, 31]. These mechanisms lead to the degradation of the material and to microcracks on the surface [15, 16]. These microcracks in turn accelerate the degradation process, resulting in by‐products that appear, for example, in a slimy state on the surface. Due to the acidic pH value of the slime, it is assumed that this is lactic acid. This strong degradation of Luminy L130 is clearly shown in the derived TG curves in Figure 1A after the 9 months of aging with a shifted curve to lower temperatures. In the 9 month old stored samples, the onset of the degradation process is around 125°C, which is very close to the temperature at which the dehydration of PLA generally takes place. The explanation for the very early onset of the decomposition process can therefore be found in the slimy and therefore predominantly liquid substance on the surface, which burns more quickly than the residual PLA.

However, the investigations also showed that if both the crystallinity value and the moisture content increase moderately and evenly, the curves do not change regardless of the storage period. This is marked in black in Figure 1B. The Luminy L130 shows a small and similar change in crystallinity value and moisture content within the first 3 months of aging. In the TG curve, this is reflected in an almost identical progression of both curves. In conclusion, it can be stated that the changes in properties such as the degree of crystallinity and moisture content of the material are reflected in the investigations of the pyrolysis process, represented by the TG curves of all three PLA types.

### 4.2 Activation Energy‐Analysis

In general, assessing the activation energy for the degradation of PLA is not a novel concept. Numerous studies have already measured this parameter using various techniques. Recent investigations, in particular, tend to use kinetic data rather than mechanical data as the basis for their analyses [23]. Table 3 provides an overview of some of the evaluated activation energies and the references. The activation energy values reported in these studies range from 97 to 164 kJ mol^−1^ [32, 33, 34].

**TABLE 3:** Activation Energy based on kinetic parameters according to literature.

Material	Reference	Method	E_a_ in kJ/mol
PLA	Alhulaybi et al. [32]	Friedman	97
PLA	Alhulaybi et al. [32]	FWO	109
PLA	Alhulaybi et al. [32]	KAS	104
PLA	Alhulaybi et al. [32]	Starink	104
PLA	Tai et al. [34]	Friedman	161.75
PLA	Mróz et al. [33]	Kissinger	152 ± 30
PLA	Mróz et al. [33]	Friedmann	164 ± 24
PLA	Mróz et al. [33]	Ozawa	155 ± 15

The fluctuations listed can be attributed to various influencing factors. In addition to the specific material type and experimental conditions, the calculated results are inevitably influenced by the model used. Consequently, the comparison and selection of an appropriate model were central aspects of this study.

#### 4.2.1 The Correlation Between Activation Energy and the Conversion Rate

In the comparative analysis of various kinetic models, explicit reference should be made to the guidelines provided by the kinetic committee of ICTAC. The committee emphasizes that activation energy values, when determined through isoconversional methods for a single‐step reaction, should ideally remain constant within the range values of the whole conversion [19, 32]. Kissinger's model assumes precisely this linear relationship and therefore provides a conversion‐independent activation energy for a single‐stage degradation process [25, 35, 36].

However, the degradation mechanism of PLA is often described as a multi‐stage process, both in the literature and in already published, own investigations [31, 37, 38, 39]. Teixeira's work summarises various theories on this subject by depicting and analysing the progression of various material parameters over the degradation cycle of PLA [31].

Assuming a multistage degradation process, alternative models such as KAS, FWO and Friedmann are better suited for the analysis. Here, complex kinetics become apparent through fluctuations in the activation energy over conversation rate [32, 33, 40].

**FIGURE 4:**
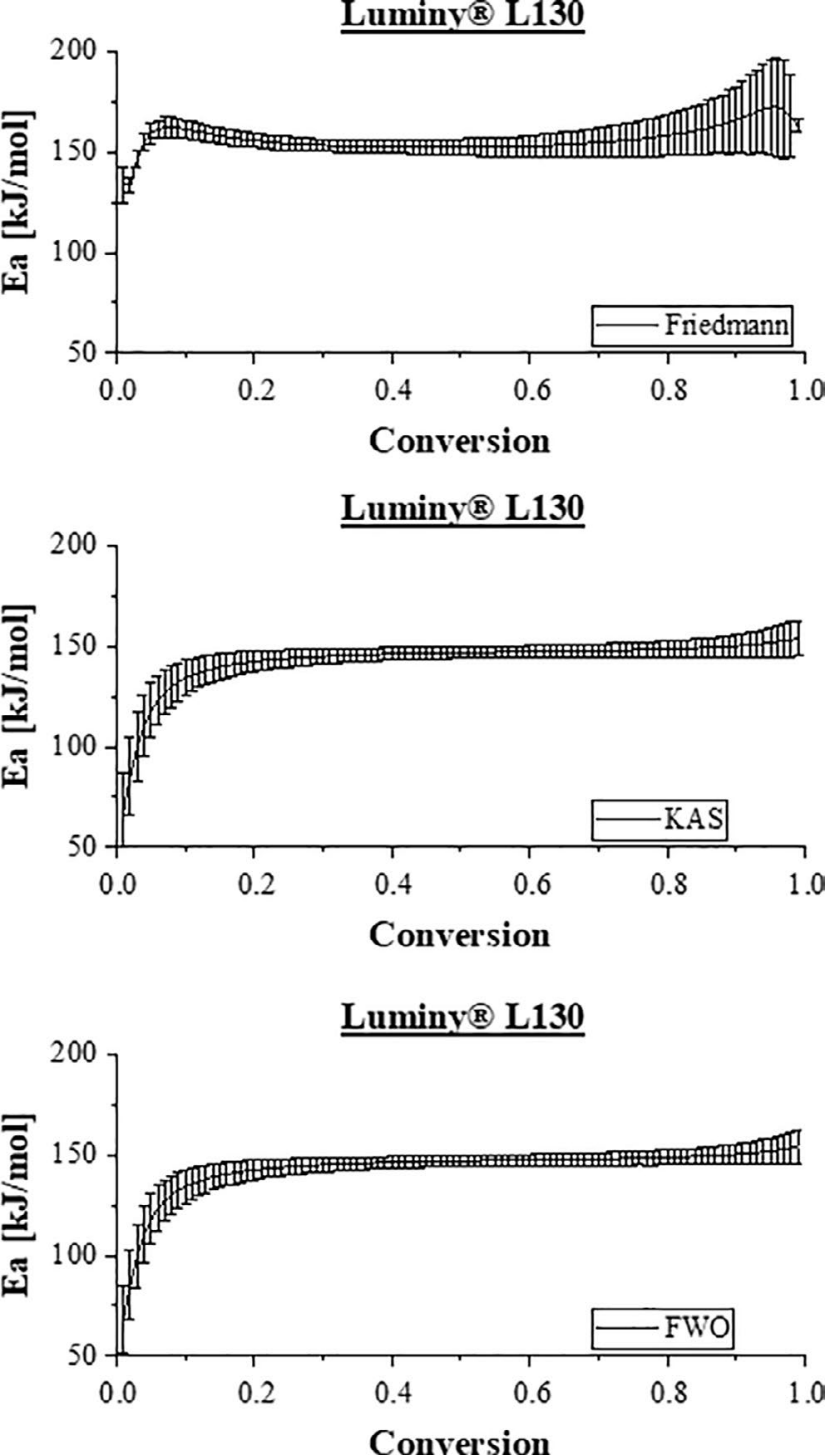
Dependence of the activation energy on the conversion rate using the example of Luminy L130 according to the FWO (*R* ^2^ = 0,98,137), Friedmann (*R* ^2^ = 0,99,995), KAS (*R* ^2^ = 0,97,123). The graphical representations generated through the KAS and FWO methodologies exhibit complete overlap.

Figure 4 illustrates the described, model‐dependent relationship between activation energy and degree of conversion using the example of unaged Luminy L130 samples. The data shown were determined using the Netzsch kinetics program. In particular, the range between 0.01 and 0.99 was screened with a step height of 0.01.

Literature mostly suggests that the correlation between activation energy and conversion rate can be categorized into two distinct ranges [33, 37, 41]. In the first range, extending from 0 to approximately a_1_ = 0.45, a noticeable elevation in activation energy is evident [33, 41]. This observed tendency is also recognized to a greater or lesser extent in this study, as can be seen in Figure 4. Concerning Luminy L130, a distinct rise in activation energy (as per FWO, KAS and Friedmann methods) is evident within the range up to approximately a_1_ = 0.1. In the second range (a_2_ > a_1_), a nearly consistent value for activation energy prevails, a phenomenon substantiated by findings in the study conducted by Mroz and Li [33, 41]. An almost consistent activation energy (150 kJ/mol) within the second timeframe can also be discerned from the presented data. In accordance with own measurements and Friedmann's analysis, a third phase emerges beyond a conversion factor of 0.8, marked by a renewed increase. The other two models FWO and KAS also show this increase to a very small extent.

All in all, each of the profiles shown, irrespective of the model used, indicate a multi‐step degradation process. Although the average activation energy shows minimal variation, the models exhibit slight discrepancies at low and very high conversion rates. Specifically, the Friedmann model implies that the pyrolytic degradation of PLA may require division into more than two stages. In comparing the determination coefficient *R* ^2^, it becomes evident that the Friedmann model best replicates the measured values. According to the literature, the differential Friedmann model is more accurate than the integral KAS and FWO models because it directly uses experimental data without assuming a specific reaction function. By employing the differential method, it captures small changes in reaction rates more precisely, making it better suited for complex and multi‐step reactions. However, this increased accuracy requires high‐quality data, as the model is more sensitive to experimental noise and errors. Despite independently conducted analyses, completely fluctuation‐free data recording cannot be guaranteed. Minor measurement deviations between different samples are documented through repeat measurements. Consequently, it would be advisable to divide the degradation process into at least two segments and assign specific activation energies to each.

The results for NP HT 202 and NP HT 203 also indicate a multi‐step degradation process. For both samples, a division into at least two distinct segments can be made, regardless of the chosen model. While NP HT 202 shows a plateau (120 kJ/mol for FWO and KAS, 140 kJ/mol for Friedmann) between 0.6 and 0.95, NP HT 203 exhibits a nearly constant value for E_a_ over the conversion rate range of 0.15–0.85, which can be documented with 100 kJ/mol for FWO and KAS, 110 kJ/mol for Friedmann. Thus, the boundaries shift slightly.

Regardless of the PLA type, the different stages of thermal degradation can be attributed to specific structural characteristics of the material. Notably, there is an initial increase in the crystalline content of the material structure, which can be attributed to thermal treatment. The α‐crystal structures formed by annealing are stable and have high melting temperatures, thereby contributing to the initial rise in activation energy. Simultaneously, the less thermally stable amorphous regions degrade. The moisture and volatile components present in the material, which are expected to evaporate within this temperature range, should also be considered.

Following this phase of reorganization, a stable plateau is achieved. A further increase in temperature eventually leads to the degradation of the crystalline regions. The subsequent rise in E_a_ can be explained by the presence of residuals, which were clearly observed in TGA measurements. For Luminy L130, residues of 1.3 m.% were documented, while for NP HT 202 and NP HT 203, 10 m.% and 11.5 m.% of the initial mass remained even at 800°C, respectively. It is important to note that NP HT 202 and NP HT 203 contain additives that can further alter the mechanisms within the material structure and influence the degradation process.

The presented results invalidate the assumption of a single‐step degradation process for PLA under thermal stress. To demonstrate that these results are not only applicable to thermal degradation but also transferable to material degradation in real‐world applications, changes in the activation energy were also determined over the course of artificially accelerated aging.

#### 4.2.2 The Correlation Between Activation Energy and the Aging of Materials

**FIGURE 5:**
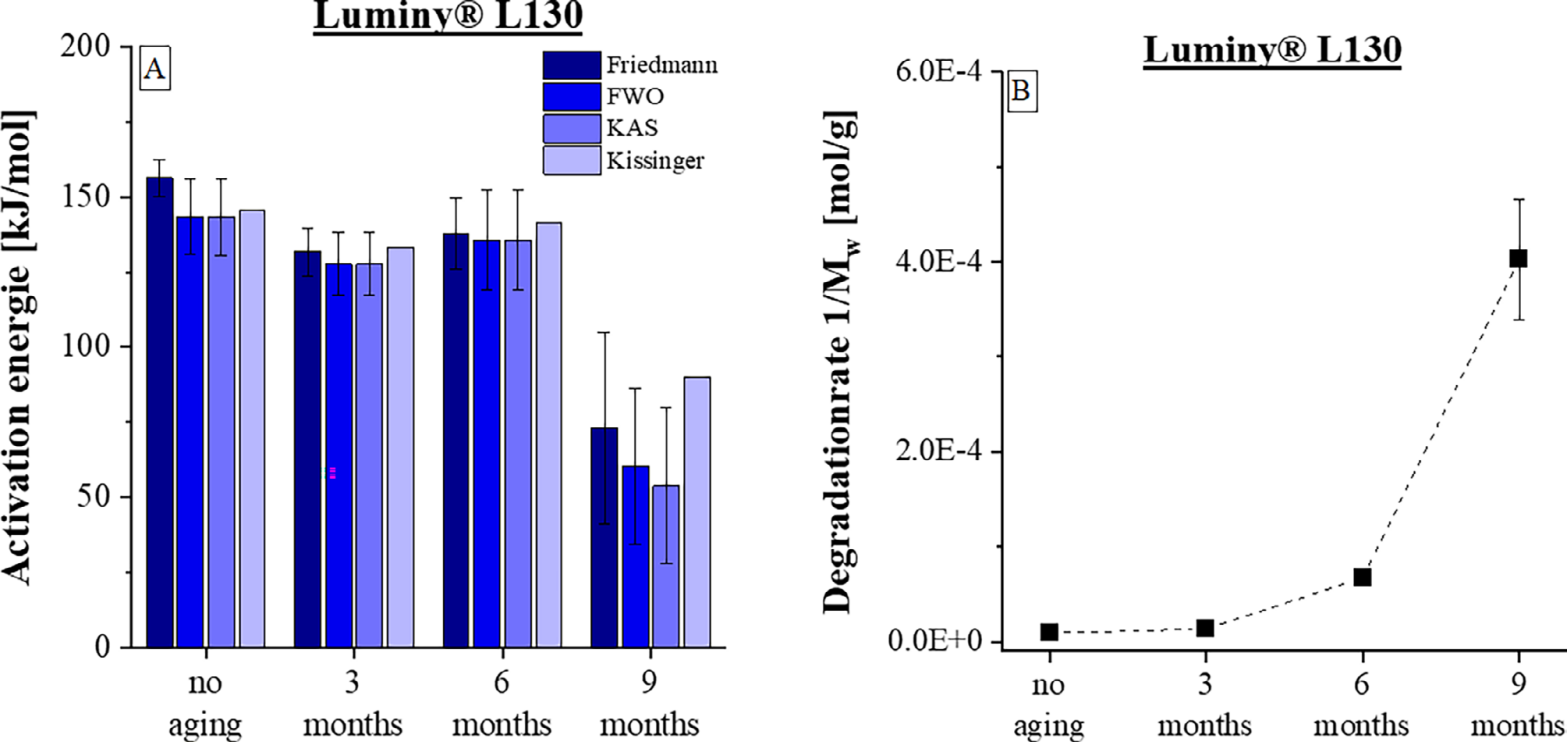
(A) Activation energies of Luminy L130 determined in the course of ageing according to the respective kinetic models. (B) Degradation rate (1/M_w_) during the ageing process according to GPC Data [37].

Specifically, the variation in material‐specific activation energies for thermal degradation during the artificially accelerated aging of PLA was analyzed using the previously discussed kinetic models. Four different aging stages were compared, and the results are presented in Figure 5 Unlike the thermal degradation described earlier, material degradation during storage in this context is primarily due to hydrolytic processes [37].

As illustrated in Figure 5A, the activation energy during the aging process at 50°C and 50% relative humidity initially changes only slightly. Samples aged for 3 and 6 months under accelerated conditions exhibit activation energies within the fluctuation range of unaged samples or slightly below. This can be explained by the fact that various parameter changes overlap, each exerting an individual influence on the activation energy. For example, own studies have shown a distinct reduction in average molecular weight during the first 2 years, which decreases material stability. In Addition, an increase in crystallinity has been observed, which contributes to enhanced thermal resistance [37]. The parameters and experimental procedure can be found within this cited paper. In the first aging time, these and all other effects resulting from the storage seem to approximately balance each other out.

The substantial decrease in activation energy (Figure 5A), associated with the increase in degradation rate (Figure 5B), can be attributed to reaching a threshold value or inflection point. For Luminy L130, this point is reached between two and 3 years under accelerated conditions. Further investigations have shown that within this period, the hydrolytic cleavage of polymer chains progresses to the extent that the amorphous regions are completely depleted [37]. As depicted in Figure 1A, the material's crystallinity reaches a maximum and subsequently declines. In contrast, the molecular weight consistently decreases during this time. Additionally, an abrupt increase in moisture uptake has been documented within this period, supporting the previous hypothesis. The material structure appears to have lost all stability and resistance to degradation. The prevailing effects can no longer counterbalance each other. Instead, there is a pronounced acceleration of the degradation process. Consequently, the material's resistance and activation energy undergo a dramatic reduction.

As shown in Table 4, the other examined PLA materials also exhibit an initial reduction in activation energy during the aging process, which is independent of the isoconversion model used. A comparison of these results indicates that Luminy L130 exhibits the highest energy threshold (> 140 kJ/mol) for thermal degradation in the unaged state, regardless of the model selected. For NP HT 203, the lowest activation energies were determined, averaging between 96 and 124 kJ/mol. Notably, the Friedmann model shows virtually no difference in the initial energy threshold between NP HT 202 and NP HT 203. A possible influence of the additives (Acetyltributylcitrate) detected in NP HT 202 and NP HT 203 should be taken into account in the interpretation.

**TABLE 4:** Development of the activation energies during aging (at 50°C and 50% relative humidity) of all investigated materials according to the selected kinetic models.

Material	Model	Activation energy in kJ/mol							
		No aging		3 months		6 months		9 months	
Luminy L130	FWO	143.33	±13	127.60	±11	135.67	±17	60.32	±26
	Friedmann	156.37	±6	131.71	±8	137.75	±12	72.98	±32
	KAS	143.20	±13	127.23	±11	135.54	±17	53.79	±26
	Kissinger	146.56		133.25		141.35		90.03	
NP HT 202	FWO	108.63	±22	87.49	±6	97.24	±24	82.78	±22
	Friedmann	125.41	±22	82.40	±9	107.44	±22	87.52	±37
	KAS	108.46	±22	87.32	±6	97.07	±24	82.59	±22
	Kissinger	134.31		81.06		93.56		90.21	
NP HT 203	FWO	96.52	±24	95.24	±12	64.93	±13	99.31	±34
	Friedmann	124.29	±90	108.13	±15	73.81	±14	102.04	±25
	KAS	96.34	±24	94.97	±12	64.70	±13	99.13	±34
	Kissinger	108.15		97.40		34.59		101.57	

It is conceivable that Acetyltributylcitrate initially reduces the activation energy, as the plasticizer reduces the intermolecular forces between the PLA chains, which could explain the evident lower *E*_a_ compared to Luminy L130. Additionally, phase separation and diffusion of Acetyltributylcitrate within the polymer can cause a non‐homogeneous distribution of the plasticizer, influencing degradation kinetics and activation energy differently. Regions with higher concentrations of plasticizer may exhibit lower activation energy values, whereas regions without plasticizer may have higher values.

Based on these results, it becomes evident why the prediction of material lifespan can only be considered an approximation. Since the material condition and thus the activation energy change during real‐world storage, the Q_10_‐factor must be continuously recalculated and adjusted. Therefore, an accurate prediction of material lifespan necessitates a dynamic adjustment of the Q_10_‐factor in accordance with changes in activation energy. Determining and using a material‐specific parameter would likely significantly improve the prediction compared to the current standard as per ASTM 1980.

## 5 Conclusion

This study investigated the thermal stability of three different PLA types over a 9 month aging period under conditions of 50°C and 50% relative humidity. The results showed a decrease in thermal stability, indicated by a shift of the derived mass loss curves to lower temperatures measured by TGA. This behavior is explained by changes in crystallinity, which initially increases but rapidly decreases with prolonged aging. The breakdown of crystalline regions increases the proportion of amorphous areas, making the material more susceptible to hydrolysis, as confirmed by increased water absorption measurements. The degradation process starts on the surface, especially below the glass transition temperature, leading to explicit changes such as the formation of a slimy layer on the surface of Luminy L130 after 9 months. The study also highlights that if crystallinity and moisture content increase moderately, the thermal degradation curves remain unchanged.

Kinetic analyses using models like KAS, FWO, and Friedmann indicate a multi‐step degradation process. The activation energy fluctuates with the conversion rate, suggesting complex kinetics. During artificial aging, a notable decrease in activation energy and increased degradation rate were observed, particularly after two to 3 years, indicating a critical point where material stability dramatically declines. This study indicates that the activation energy for the pyrolytic degradation of PLA varies with the degree of conversion and aging time, highlighting a complex degradation mechanism. The activation energy values obtained using various isoconversional methods showed a consistent trend, suggesting reliable and reproducible results. These findings align with the notion that PLA undergoes a multi‐step degradation process, influenced by its thermal history and aging conditions. Previous studies have reported activation energy values for PLA degradation ranging from 100 to 200 kJ/mol, depending on the experimental setup and conditions. The results presented here fall within the expected range, providing a detailed activation energy profile across different conversion rates and aging stages. This nuanced analysis enhances the prediction models for PLA degradation in various applications. When calculating a PLA‐specific Q_10_‐factor to optimize artificially accelerated aging in accordance with ASTM 1980, it is crucial to acknowledge that realistic aging simulation requires dynamic adjustment of the Q_10_‐factor to reflect the current material condition. The results underscore the changes in activation energy in relation to complex material transformations. Furthermore, it should be noted that assuming an average activation energy value can still significantly improve the accuracy of degradation forecasts. Determining the activation energy can therefore be highly beneficial. The KAS, FWO or even better the Friedmann model should be used therefore.

## Author Contributions

Margarita Reit and Natalie Krug: conceptualization, methodology, formal analysis, investigation, data curation, writing – original draft preparation, visualization. Hans‐Peter Heim: resources, funding acquisition. Hans‐Peter Heim and Jan‐Christoph Zarges: writing – review and editing, supervision, project administration. All authors have read and agreed to the published version of the manuscript.

## Conflicts of Interest

The authors declare no conflicts of interest.

## Data Availability

The data that support the findings of this study are available on request from the corresponding author. The data are not publicly available due to privacy or ethical restrictions.
